# Impact of multimorbidity on the first ts/bDMARD effectiveness and retention rate after two years of follow-up in patients with rheumatoid arthritis from the BIOBADASER registry

**DOI:** 10.1186/s13075-024-03287-9

**Published:** 2024-02-23

**Authors:** Jerusalem Calvo-Gutiérrez, Clementina López-Medina, Lucía Otero-Varela, Alejandro Escudero-Contreras, Rafaela Ortega-Castro, Lourdes Ladehesa-Pineda, Cristina Campos, Pilar Bernabeu-Gonzalvez, Ana Pérez-Gómez, Alicia García-Dorta, Dolores Ruiz-Montesino, Manuel Pombo-Suarez, Inmaculada Ros-Vilamajo, Fernando Sánchez-Alonso, Isabel Castrejón

**Affiliations:** 1 Rehabilitation Department, Infanta Margarita University Hospital, Cabra, Cordoba, Spain; 2 Maimonides Institute for Biomedical Research of Cordoba (IMIBIC), Cordoba, Spain; 3 Rheumatology Department, Reina Sofia University Hospital, Menendez Pidal Avenue, s/n. 14004, Cordoba, Spain; 4 Medical and Surgical Sciences Department, University of Cordoba, Cordoba, Spain; 5 Research Unit, Spanish Foundation of Rheumatology, Madrid, Spain; 6 Rheumatology Department, Hospital General Universitario de Valencia, Valencia, Spain; 7 Rheumatology Department, Hospital General Universitario Dr. Balmis, ISABIAL, Alicante, Spain; 8 Rheumatology Department, Hospital Universitario Príncipe de Asturias, Madrid, Spain; 9 Rheumatology Department, Hospital Universitario de Canarias, Canarias, Spain; 10 Rheumatology Department, Hospital Universitario Virgen de la Macarena, Sevilla, Spain; 11 Rheumatology Department, Hospital Clínico Universitario de Santiago, A Coruña, Spain; 12 Rheumatology Department, Hospital Universitario Son Llatzer, Baleares, Spain; 13 Rheumatology Department, Gregorio Marañón University Hospital, Madrid, Spain

**Keywords:** Rheumatoid arthritis, Comorbidities, bDMARDs

## Abstract

**Background:**

Patients with Rheumatoid Arthritis (RA) have a higher prevalence of comorbidities compared to the general population. However, the implications of multimorbidity on therapeutic response and treatment retention remain unexplored. Objectives: (a) To evaluate the impact of multimorbidity on the effectiveness of the first targeted synthetic or biologic disease-modifying antirheumatic drug (ts/bDMARD), in patients with RA after 2-year follow-up; (b) to investigate the influence of multimorbidity on treatment retention rate.

**Methods:**

Patients with RA from the BIOBADASER registry exposed to a first ts/bDMARDs were included. Patients were categorized based on multimorbidity status at baseline, defined as a Charlson Comorbidity index (CCI) score ≥ 3. A linear regression model, adjusted for sex and age, was employed to compare the absolute DAS28 score over time after ts/bDMARD initiation between the two groups. The Log-Rank test and Kaplan-Meier curve were used to compare the retention rates of the first ts/bDMARD between the groups.

**Results:**

A total of 1128 patients initiating ts/bDMARD were included, with 107 (9.3%) exhibiting multimorbidity. The linear regression model showed significantly higher DAS28 (beta coefficient 0.33, 95%CI:0.07–0.58) over a two-year period in patients with multimorbidity, even after adjusting for age and sex. Finally, no differences in the ts/bDMARD retention rate were found between groups (median 6.94–6.96 years in CCI < 3 vs. 5.68–5.62 in CCI ≥ 3; *p* = 0.610).

**Conclusions:**

Multimorbidity in patients with RA was associated with greater DAS28 scores within the first two years after ts/bDMARD initiation, in comparison with patients without multimorbidity. A slightly shorter retention rate was found in patients with multimorbidity, although the difference was non-significant.

**Supplementary Information:**

The online version contains supplementary material available at 10.1186/s13075-024-03287-9.

## Background

Rheumatoid arthritis (RA) is a chronic inflammatory disease that primarily affects the joints, leading to inflammation of the synovium and cartilage damage [1]. The most common symptoms include pain, swelling, and stiffness in the affected joints. Additionally, patients with RA may also develop extraarticular manifestations (such as vasculitis and pulmonary involvement) and systemic comorbidities, defined as the existence or occurrence of any distinct additional entity during the clinical course of a patient who has RA [2]. Comorbidities in patients with RA can arise either as a consequence of the rheumatic treatment (e.g., non-steroidal anti-inflammatory drugs inducing hypertension) or be associated directly with RA itself. These comorbid conditions pose challenges in managing the rheumatic disease affecting their treatment in various ways, such as contraindications for certain drugs, perpetuation of the inflammation and treatment non-adherence [3, 4]. In fact, comorbidities are recognized as significant factors contributing to the difficult-to-treat (D2T) state in patients with RA, impacting their quality of life and potentially limiting RA treatment options. Recently, the EULAR Task Force convened to develop points to consider for the management of D2T RA, emphasizing the need for cautious interpretation of composite indices and clinical evaluation when comorbidities are present, since these conditions may lead to an overestimation of disease activity [5].

Although various comorbidities can influence the patient reported outcomes (PROs), the existing evidence primary focuses on obesity and fibromyalgia [6–8]. A recent study showed that obese patients with RA are less likely to have true synovitis accurately represented by a clinically assessed swollen joint compared to measurements obtained by ultrasound. As a result, disease activity in obese RA patients might be overestimated when using the clinical disease activity index (CDAI) or the disease activity score 28 (DAS28) calculations [7]. In a recent meta-analysis examining the influence of fibromyalgia on disease activity, patients with this comorbid condition were found to have higher DAS28 scores compared to those with RA alone [8]. However, there are limited studies that have explored the impact of multimorbidity (i.e., defined as the coexistence of two or more chronic health conditions in addition to the RA [9]) on treatment efficacy and adherence in RA patients. Previous studies observed a less pronounced enhancement in the quality of life among RA patients with high Charlson Comorbidity Index (CCI) levels [10]. Conversely, other studies suggested that CCI scores were not associated with early discontinuation of Tumour Necrosis Factor (TNF) inhibitors [11]. These limited and contradictory findings emphasize the necessity for more extensive research on the impact of comorbidities, particularly multimorbidity, on drug efficacy and persistence of treatment in RA patients.

BIOBADASER is a national, prospective, and multicenter Spanish registry on adverse events of targeted synthetic and biological disease-modifying anti-rheumatic drugs (ts/bDMARDs) in rheumatic diseases. This comprehensive registry presents a unique opportunity to evaluate the impact of multimorbidity on treatment effectiveness and adherence to ts/bDMARDs in sizable cohort of patients with RA. This study aims to achieve two objectives: (a) to evaluate the impact of multimorbidity on the first ts/bDMARD’s effectiveness in patients with RA after a 2-year follow-up period, and (b) to determine the influence of these comorbidities on the first ts/bDMARD’s retention rate.

By investigating these aspects, this research seeks to enhance our understanding of how multiple health conditions affect the response to ts/bDMARDs and the long-term adherence to these treatments among patients with RA. The findings could provide valuable insights for optimizing treatment strategies and improving outcomes for RA patients with multimorbidity.

## Methods

### Study design

This is a retrospective analysis conducted using real-world data from the BIOBADASER nationwide safety registry, which comprises patients with rheumatic diseases undergoing treatment with bDMARDs (including biosimilars) and tsDMARDs. Stablished in 2001 and promoted by the Spanish Foundation of Rheumatology, BIOBADASER has consistently collected data from routine clinical practice, which is then monitored yearly for quality control. The collection of comorbidity data started when the third phase of the registry was launched in 2015, and thus, the information presented in this manuscript includes data from BIOBADASER phase III until October 2022, when the data is locked for analysis.

Ethical approval for the registry was obtained from the Hospital Clínic de Barcelona Research Ethics Committee (FER-ADA-2015-01), while the current reference committee is the Hospital de Canarias. All participants willingly signed an informed consent to participate in the BIOBADASER registry

### Population

For this study, we included adult patients (≥ 18 years) diagnosed with RA based on their clinician’s assessment and fulfilling the ACR/EULAR 2010 classification criteria [12]. Moreover, we specifically focused on ts/bDMARD-naïve individuals (i.e., patients who were about to initiate their first-line treatment with a ts/bDMARD).

In this analysis, we restricted our focus to the initial two years of follow-up, with one visit per year (i.e., baseline, 1-year, and 2-year visits).

### Variables

- Sociodemographic data such as age at the initiation of the ts/bDMARD and sex were collected at the baseline assessment.
- Disease-related variables, including Rheumatoid Factor (RF) positivity and anti-citrullinated peptide antibody (ACPA) status were collected.
- To assess disease activity, various measures were collected at each visit, including the tender joints count (TJC), swollen joints count (SJC) and Patient’s global health assessment (PGH) using a visual analogue scale ranging from 0 to 10. Erythrocyte sedimentation rate (ESR) in mmHg and c-reactive protein (CRP) in mg/L were monitored at each visit. In addition, the composite disease activity score 28 (DAS28) was evaluated at each time-point. Remission status was defined as a DAS28 lower than 2.6 (DAS28<2.6) [13].
- Comorbidities: we considered all the comorbidities listed in the Charlson Comorbidity Index (CCI), which is extensively described elsewhere and widely used in RA patients [14, 15]. Briefly, this index is a valuable tool for predicting 10-year survival in patients with multiple comorbidities and includes nineteen conditions: myocardial infraction, congestive heart failure, peripheral vascular disease, cerebrovascular accident or transient ischemic attack, dementia, chronic obstructive pulmonary disease (COPD), connective tissue disease, peptic ulcer disease, liver disease (mild or moderate to severe), diabetes mellitus (uncomplicated or with end-organ damage), hemiplegia, moderate to severe chronic kidney disease, solid tumor (localized or metastatic), leukemia, lymphoma and Acquired Immune Deficiency Syndrome (AIDS). Each condition is assigned a weight from 1 to 6, based on its estimated 1-year mortality. The individual weights are then summed to calculate the CCI, with scores ranging from 0 to 37, corresponding to estimated 10-year survival rates of 98% to 0%, respectively. For this analysis, we utilized a modified version of the CCI, in which is the presence of connective tissue disease is assigned one point in the scoring system. As all patients in our study were diagnosed with RA, they will all have at least a CCI score of 1. Age was not considered as an extra point in this index.
- Treatments: all patients included in this study were treatment-naïve to ts/bDMARD and were about to initiate their first-line treatment with these medications. The bDMARDs category encompassed Tumor Necrosis Factor inhibitors (TNFi), interleukin-6 inhibitors (IL-6i), CD20 inhibitors (CD20i) and Abatacept. Three patients who initiated bDMARDs not approved for RA (e.g., IL1i, and IL12/23i) were also included. The tsDMARDs category included Janus Kinases inhibitors (JAKi), such as Tofacitinib, Baricitinib, Upadactinib and Filgotinib. Data on the dates of drug initiation and withdrawal were collected to evaluate the retention rate of patients to their respective treatments.

### Statistical analysis

First, a descriptive analysis was performed using means and standard deviation (SD) for continuous variables, and absolute and relative frequencies for qualitative variables.

As per prior literature, patients with multimorbidity are characterized as individuals having two or more chronic health conditions in addition to the RA. Based on this definition, we classified patients in two groups: those with multimorbidity (i.e., CCI score ≥ 3) and those without it (CCI score < 3) [9]. Baseline demographic and clinical characteristics were compared between groups using chi-squared tests and T-test for binary and continuous variables, respectively.

To assess whether the presence of multimorbidity affects treatment effectiveness, we compared the percentage of patients achieving remission status (DAS28 < 2.6) at 1-year and 2-year timepoints between those with CCI scores ≥ 3 and < 3 CCI.

Furthermore, we conducted a linear regression model using DAS28 over the two years as dependent variable and the presence of multimorbidity as independent variable. Given the temporal variation of DAS28, we performed a regression model to panel data structure, considering random-effects models by using the Generalized Least Squares (GLS) estimator. This analysis aimed to evaluate the association between multimorbidity and the change in disease activity. To account for potential confounding factors (i.e., age at the initiation of the ts/bDMARD and sex) which may influence both the number of comorbidities and the subjective evaluation of the patient, an additional model was explored adjusting for these variables and for the timepoint. Additional multivariable linear models were conducted as sensitivity analysis to evaluate the association of multimorbidity (using CCI scores ≥ 3 and CCI as continuous variable) with the different components of the DAS28 (i.e., SJC, TJC, PGH and ESR).

Finally, we compared the retention rate of the first ts/bDMARD between the two patients’ groups (i.e., ≥ 3 vs. < 3 CCI scores) using a Kaplan-Meier curve and a log-rank test. However, in this analysis we excluded patients who received CD20 inhibitors (rituximab) given that their unique treatment regimen (which involves one infusion every 6 months) could potentially introduce bias into the results of the retention rate analysis.

All contrasts were bilateral and considered significant when the *p*-value < 0.05. Data were collected, processed, and analysed using Stata 13.1 (StataCorp®).

## Results

A total of 1128 patients were included in the analysis, out of which 860 (76.2%) were female and with a mean age of 56.0 (SD = 12.1) years (Table 1). Additionally, 75% of patients tested positive for Rheumatoid Factor and the mean DAS28 was 4.6 (1.3). TNFi were the most frequently prescribed drugs in the total population (61.4%), followed by Janus Kinase inhibitors (JAKi) at 14.7%, and CTLA4 inhibitors (9.3%).

**Table 1:** Patients’ characteristics at baseline of the included population, stratified by the presence of multimorbidity according to the Charlson Comorbidity Index score

	**Total** ***N* = 1128** **N (%)**	**Multimorbidity** **(CCI score ≥ 3)** ***N* = 105** **N (%)**	**No multimorbidity (CCI score < 3)** ***N* = 1023** **N (%)**	***p*-value**
**Age at the drug initiation, mean (SD)**	56.0 (12.1)	65.1 (9.2)	55.1 (12.0)	** < 0.001**
**Disease duration, mean (SD)**	7.3 (7.8)	10.3 (10.2)	7.0 (7.4)	** < 0.001**
**Sex (female)**	860 (76.2%)	71 (67.6%)	789 (77.1%)	**0.029**
**RF positive**	843/1099 (74.9%)	77/99 (73.3%)	766/999 (75.0%)	0.250
**ACPA positive**	244/1063 (21.7%)	24/93 (22.9%)	220/970 (21.6%)	**0.012**
**TJC**				
** - Mean (SD)**	6.1 (5.7)	6.2 (5.5)	6.1 (5.8)	0.948
** - Median (IQR)**	5.0 [2.0–8.0]	4.5 [2.0–8.5]	5.0 [2.0–8.0]	0.803
**SJC**				
** - mean (SD)**	4.2 (4.3)	5.9 (5.2)	4.0 (4.1)	** < 0.001**
** - Median (IQR)**	3.0 [1.0–6.0]	4.0 [2.0–8.0]	3.0 [1.0–5.0]	** < 0.001**
**PGH**				
** - Mean (SD)**	5.8 (2.2)	5.6 (2.2)	5.9 (2.2)	0.382
** - Median (IQR)**	6.0 [5.0–7.0]	6.0 [5.0–7.0]	6.0 [5.0–7.0]	0.401
**ESR (mm/h)**				
** - Mean (SD)**	28.9 (23.9)	41.7 (29.9)	27.6 (22.8)	** < 0.001**
** - Median (IQR)**	22.0 [11.0–40.0]	35.0 [17.0–59.0]	21.5 [10.0–38.0]	** < 0.001**
**CRP (mg/L)**				
** - Mean (SD)**	7.6 (14.7)	13.5 (20.4)	7.1 (13.9)	** < 0.001**
** - Median (IQR)**	2.5 [0.7–8.1]	3.7 [1.3–20.2]	2.4 [0.7–7.8]	**0.002**
**DAS28**				
** - Mean (SD)**	4.6 (1.3)	5.0 (1.3)	4.6 (1.3)	**0.002**
** - Median (IQR)**	4.6 [3.8–5.4]	5.1 [4.2–5.9]	4.6 [3.8–5.4]	
**Treatment**				
** - TNF inhibitors**	693 (61.4%)	40 (38.1%)	653 (63.8%)	** < 0.001**
** - IL6 inhibitors**	106 (9.4%)	10 (9.5%)	96 (9.4%)	
** - CD20 inhibitors**	55 (4.9%)	12 (11.4%)	43 (4.2%)	
** - JAK inhibitors**	166 (14.7%)	19 (18.1%)	147 (14.4%)	
** - CTLA4 inhibitors**	105 (9.3%)	23 (21.9%)	82 (8.0%)	
** - IL12/23 inhibitors**	2 (0.2%)	1 (1.0%)	1 (0.1%)	
** - IL1 inhibitors**	1 (0.1%)	0 (0%)	1 (0.1%)	
**Charlson Comorbidity index (mean), median [IQR]**	1.0 [1.0—1.0]	3.0 [3.0—4.0]	1.0 [1.0—1.0]	** < 0.001**

Available data for the overall population: TJC = 984, SJC = 986, PGH = 965, ESR = 995, CRP = 957, DAS28 = 1128

Available data for multimorbidity group: TJC = 92, SJC = 93, PGH = 91, ESR = 93, CRP = 85, DAS28 = 105

Available data for no multimorbidity group: TJC = 892, SJC = 893, PGH = 874, ESR = 902, CRP = 872, DAS28 = 1023

*ACPA* anti-citrullinated peptide antibody, *CRP* c-reactive protein, *DAS28* disease activity score 28, *ESR* erythrocyte sedimentation rate, *IQR* Interquartile Range, *SJC* Swollen Joint Count, *TJC* Tender Joint Count, *PGH* Patient Global Health, *RF* Rheumatoid Factor

The most prevalent comorbidities among the patients were uncomplicated Diabetes Mellitus (*n* = 59, 5.2%), localized solid tumor (*n* = 55, 4.9%) and COPD (*n* = 35, 3.1%) (Table 2).

**Table 2:** Prevalence of the comorbidities included in the Charlson Comorbidity Index score

**Comorbidity**	**Total = 1128** **N (%)**	**Missing** **N**
Myocardial infraction	17 (1.5%)	1
Congestive heart failure	13 (1.2%)	0
Peripheral vascular disease	26 (2.3%)	3
Cerebrovascular accident or transient ischemic attack	10 (0.9%)	3
Dementia	1 (0.1%)	3
Chronic obstructive pulmonary disease (COPD)	35 (3.1%)	3
Peptic ulcer disease	14 (1.2%)	3
Liver disease (mild)	22 (2.0%)	3
Liver disease (moderate to severe)	2 (0.2%)	3
Diabetes mellitus (uncomplicated)	59 (5.2%)	0
Diabetes mellitus (with end-organ damage)	7 (0.6%)	0
Hemiplegia	0 (0.0%)	3
Chronic kidney disease (moderate - severe)	21 (1.9%)	6
Solid tumor (localized)	55 (4.9%)	3
Solid tumor (metastatic)	1 (0.1%)	3
Leukemia	1 (0.1%)	4
Lymphoma	3 (0.3%)	4
Acquired Immune Deficiency Syndrome (AIDS)	0 (0.0%)	4

A total of 105 (9.3%) of patients suffered from multimorbidity (CCI score ≥ 3) and 1023 (90.7%) did not suffer this condition (CCI score < 3). Those with multimorbidity demonstrated an older age at the moment of the drug initiation (65.1 (SD = 9.2) vs. 55.1 (SD = 12.0) years), lower proportion of females (67.6% vs. 77.1%) and higher disease activity at baseline (DAS28 5.0 (SD = 1.3) vs. 4.6 (SD = 1.3)) in comparison with patients with a CCI score < 3. Furthermore, differences were observed in the prescribed medications between both groups (Table 1).

### Association between multimorbidity and the effectiveness of ts/bDMARD

The percentage of patients achieving remission (DAS28 < 2.6) after 1 year of follow-up was 44.6% for patients with multimorbidity (CCI score ≥ 3) and 50.5% for patients without this condition (CCI score < 3). However, this difference was not statistically significant (*p* = 0.279). Similarly, after 2 years of follow-up, comparable rates of remission were observed, with 41.1% of patients with a CCI score of ≥ 3 and 53.2% of patients with a CCI score < 3 CCI achieving remission (Table 3).

**Table 3:** Proportion of patients achieving remission according to the presence of multimorbidity

	**Multimorbidity** **(CCI score ≥ 3)** **N (%)**	**No multimorbidity** **(CCI score < 3)** **N (%)**	***p*-value**
**DAS28 < 2.6 at baseline**	7/105 (6.7%)	60/1023 (5.9%)	0.741
**DAS28 < 2.6 at 1-year**	41/92 (44.6%)	479/949 (50.5%)	0.279
**DAS28 < 2.6 at 2-year**	23/56 (41.1%)	300/564 (53.2%)	0.083

*CCI* Charlson Comorbidity Index, *DAS28* disease activity score 28

### Association between multimorbidity and DAS28

The association between the presence of multimorbidity and the DAS28 over a two-year period was assessed using linear regression, as detailed in Table 4. Patients with multimorbidity (CCI score ≥ 3) had higher DAS28 values compared to those without this condition (CCI score < 3). Specifically, patients with multimorbidity showed an increase in the DAS28 of 0.37 points (95%CI: 0.17–0.57) when compared to those without multimorbidity and, even after adjusting for confounding factors, this increase remained significant (adjusted beta coefficient 0.33 (95%CI: 0.07–0.58)).

**Table 4:** Association between the change in DAS28 score and the presence of multimorbidity according to the Charlson Comorbidity Index score

	**Crude linear regression**		**Adjusted linear regression**	
	**Beta coefficient (95%CI)**	***p*-value**	**Beta coefficient (95%CI)**	***p*-value**
**Multimorbidity (CCI score ≥ 3)**	0.37 (0.17 to 0.57)	0.002	0.33 (0.07 to 0.58)	0.011
**Sex (female)**	-	-	0.35 (0.18 to 0.52)	< 0.001
**Age at the drug initiation**	-	-	0.01 (0.01 to 0.02)	< 0.001
**1-year timepoint**	-	-	-1.45 (-1.82 to -1.09)	< 0.001
**2-year timepoint**	-	-	-1.79 (-2.22 to -1.35)	0.001

*CCI* Charlson Comorbidity Index, *95%CI* 95% Confidence Interval, *DAS28* disease activity score 28

The sensitivity analysis using the different components of the DAS28 showed an association between the presence of multimorbidity and SJC (adjusted beta coefficient 1.77 (95%CI 0.86 to 2.67)) and the ESR (adjusted beta coefficient 11.72 (95%CI 6.70 to 16.75)) after adjusting for confounding factors. Similarly, the CCI as a continuous outcome was significantly associated with the DAS28 score (adjusted beta coefficient 0.11 (95%CI 0.03 to 0.20)). No associations were found with TJC or PGH (Supplementary Tables 1 and Supplementary Table 2). Furthermore, the choice of drug did not affect the association between multimorbidity and DAS28 (data not shown).

### Impact of the presence of multimorbidity on the adherence to the first ts/bDMARD

**Fig. 1:**
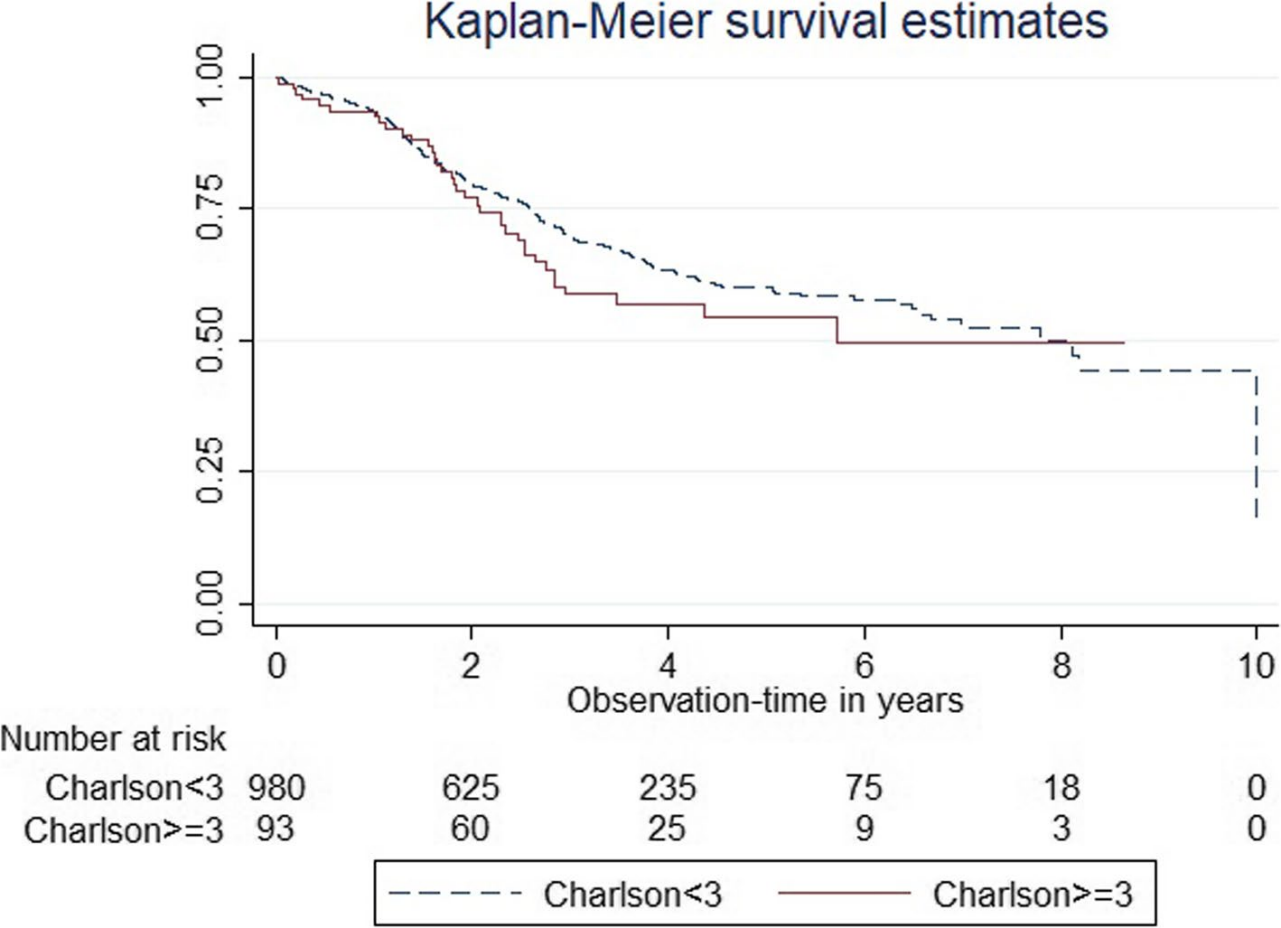
First ts/bDMARD retention rate based on the presence of multimorbidity according to the Charlson Comorbidity Index score

The comparison of retention rates for the initial ts/bDMARD (excluding Rituximab) between the two groups is represented in Fig. 1. The median retention rate for patients with multimorbidity (CCI score ≥ 3) was determined to be in between 6.94 and 6.96 years, while for those without multimorbidity (CCI score < 3), it was between 5.68 and 5.62 years. There were no significant differences observed across the treatment groups in relation to these retention rates (*p* = 0.610).

## Discussion

This study conducted in the Spanish BIOBADASER registry suggests that the presence of multimorbidity (defined as CCI score ≥ 3) in patients with RA is associated with higher levels of disease activity over the first two years of follow-up after the initiation of a ts/bDMARD in first line, in comparison with patients without multimorbidity. These findings indicate a lower likelihood of achieving disease activity control among patients with RA and concomitant comorbidities after the introduction of ts/bDMARD treatment. Nevertheless, the presence of multimorbidity do not appear to exhibit an association with either a diminished rate of remission or a shortened retention rate, when compared to patients without this condition.

Numerous studies have assessed the impact of single comorbidities (such as fibromyalgia or obesity) on treatment effectiveness. However, limited research has explored the effect of multiple comorbid conditions over time. This gap in knowledge prompted our focus on the concept of multimorbidity, defined as the presence of two or more chronic health conditions in addition to the RA.

Within this cohort, only 9.3% of patients showed multimorbidity, signifying a notably lower percentage when compared with other cohorts where the prevalence of this condition ranges between 30 and 60% [9, 15]. This discrepancy can likely be attributed to two underlying factors. Firstly, the divergent indices employed to assess comorbidities. Notably, the BIOBADASER study utilizes the CCI, encompassing nineteen distinct conditions, whereas other studies employ the counted multimorbidity index (cMMI) including forty different morbid conditions. This variance inevitably leads to an augmented prevalence of the multimorbidity status in the latter cases. Furthermore, the incongruity of the prevalence of multimorbidity could also be attributed to the distinctive characteristic inherent to the BIOBADASER cohort. This particular cohort incorporates patients at ts/bDMARDs initiation, meaning that these patients possess an optimal health status for commencing this type of drugs. In fact, it has been demonstrated that patients with RA and high number of chronic conditions are less likely to receive ts/bDMARDs in comparison with patients without comorbidities [16].

At baseline, multimorbid patients exhibited a higher prevalence of males and advanced age in comparison with those without multimorbid condition. For this reason, we decided to adjust the linear regression models by these variables, since these factors may influence both the number of comorbidities and the patient global scale. We found that the presence of a multimorbid condition was significantly associated with a higher level of disease activity (as indicated by DAS28) throughout the two-years of follow up after the initiation of a ts/bDMARD. Patients with multimorbid condition showed an average increment of 0.33 points (95%CI: 0.07–0.58) in DAS28 in comparison with patients without multimorbidity after adjusting for age and sex. This means that, in clinical practice, these patients will score higher on the disease activity questionnaires, leading to a lower likelihood of achieving disease activity control. These findings align with the points to consider for the management of D2T RA proposed by the EULAR Task Force, underlining the importance of careful interpretation of composite indices in the presence of comorbidities [5]. By presenting these results, we contribute new insights into the implications of multimorbidity, extending our understanding beyond the effects of individual comorbidities such as obesity or fibromyalgia.

We observed a similar pattern in the discontinuation of the first ts/bDMARD among patients with and without multimorbidity. The retention rate serves as a surrogate marker of both effectiveness and safety, suggesting that the treatment tolerance could be comparable between groups. However, as mentioned before, the group with multimorbidity comprises a notably limited number of patients, which potentially restricts the statistical power needed to detect significant differences in this specific analysis.

Our study has certain strengths while also acknowledging limitations. One limitation is that our assessment of comorbidities solely encompassed those present at baseline, without accounting for any potential comorbidities that may have emerged between subsequent visits. Another limitation is represented by the relatively low prevalence of patients displaying multimorbidity within this registry, which prevents to achieve enough statistical power for certain comparisons. Nonetheless, this low prevalence of multimorbidity may be explained by three key factors. Firstly, the specific profile of patients initiating a ts/bDMARD treatment could contribute to the observed low prevalence. Secondly, the population has a low average age, with a mean age of approximately 56 years at the inclusion. Thirdly, the utilization of the CCI, which accounts for only 19 comorbidities, and which excludes some common conditions such as fibromyalgia, depression, lung disease or hypertension. The non-inclusion of these frequent comorbidities in the CCI may have resulted in a lower prevalence of multimorbidity compared to previously reported studies that included these common conditions [17]. However, the CCI index was included in the BIOBADASER case report form because it is widely used and is often considered the gold standard measure to assess comorbidity in clinical research studies [18, 19]. Consequently, no other indexes such as the Rheumatic Disease Comorbidity Index (RDCI) could be tested since some of the necessary variables are missing [20]. We acknowledge that the use of the CCI in this analysis has some advantages and disadvantages. One advantage is the robustness of this index to predict long-term mortality and the smaller number of comorbidities required, making it easy to implement in clinical practice. However, this can also be seen as a disadvantage, as many important comorbidities such as depression are not covered by this index [19]. Another limitation is that only first-line treatments have been included, but this was deliberate to ensure a homogeneous population. However, considering the differences observed in treatment choices based on CCI scores, future research could investigate the potential impact of multimorbidity on treatment success rates and choice. One strength of this study resides in its prospective design, facilitating an assessment of the influence of multimorbidity on disease activity not only at a singular timepoint but also over time and subsequent to treatment initiation. Additionally, the observational nature of the analysis conducted within this cohort using real-world data augments the external validity of the findings. Finally, the quality of the collected data holds significance, as BIOBADASER is a well-known registry that incorporates online and on-site monitoring conducted by a specialized Clinical Research Associate once a year, which ensures the reliability of the results.

## Conclusion

In summary, the presence of multimorbidity in patients with RA was associated with less favourable disease activity scores after two years of follow-up after the initiation of ts/bDMARD, in comparison with patients without multimorbidity. However, no greater discontinuation of the treatment was observed in multimorbid patients. These results might offer valuable perspectives on enhancing treatment approaches and bettering results for individuals with RA and multiple health conditions. However, further prospective analysis should be conducted to comprehensively assess the impact of multimorbidity on disease outcomes and treatment effectiveness.

### Supplementary Information

**Supplementary Material 1.**

**Supplementary Material 2.**

## Data Availability

The data support the findings of this study are available from the Spanish Foundation of Rheumatology, although restrictions apply to the availability of these data, which were used under license for the current study and are therefore not publicly available. However, data are available from the authors upon reasonable request and with permission of the Spanish Foundation of Rheumatology.

## Abbreviations

ACPA: Anti-citrullinated peptide antibody
bDMARDs: Biologic disease-modifying anti-rheumatic drug
CI: Confidence Interval
CDAI: Clinical disease activity index
CRP: C-reactive protein
DAS28: Disease activity score 28
ESR: Erythrocyte sedimentation rate
IQR: Interquartile Range
JAKi: Janus Kinase inhibitors
PGH: Patient Global Health
RA: Rheumatoid Arthritis
RF: Rheumatoid Factor
SD: Standard Deviation
SJC: Swollen Joints Count
TJC: Tender Joints Count
TNFi: Tumour Necrosis Factor inhibitors
tsDMARDs: Targeted synthetic disease-modifying anti-rheumatic drug
