# Supplementary material for: Impact of multimorbidity on the first ts/bDMARD effectiveness and retention rate after two years of follow-up in patients with rheumatoid arthritis from the BIOBADASER registry
Source: Arthritis Res Ther. 2024 Feb 23;26:57. doi: 10.1186/s13075-024-03287-9 (PMC10885598; doi:10.1186/s13075-024-03287-9)
Supplement: Supplementary file 1 — Supplementary Material 1. [file 13075_2024_3287_MOESM1_ESM.docx]

**Supplementary Table 1. Association between the presence of multimorbidity according to the Charlson Comorbidity Index score and the change in the DAS28 components score.**

|  | **Adjusted linear regression**  **TJC** | | **Adjusted linear regression**  **SJC** | | **Adjusted linear regression**  **PGH** | | **Adjusted linear regression**  **ESR** | |
| --- | --- | --- | --- | --- | --- | --- | --- | --- |
|  | **Beta coefficient (95%CI)** | **p-value** | **Beta coefficient (95%CI)** | **p-value** | **Beta coefficient (95%CI)** | **p-value** | **Beta coefficient (95%CI)** | **p-value** |
| **Multimorbidity (CCI score ≥3)** | -0.2 (-1.4 to 1.1) | 0.783 | **1.8 (0.9 to 2.7)** | **<0.001** | -0.2 (-0.6 to 0.3) | 0.528 | **11.7 (6.7 to 16.8)** | **<0.001** |
| **Sex (female)** | 0.8 (-0.0 to 1.6) | 0.059 | 0.0 (-0.6 to 0.6) | 0.999 | **0.4 (0.0 to 0.7)** | **0.034** | **4.8 (1.4 to 8.2)** | **0.006** |
| **Age at the drug initiation** | 0.0 (-0.0 to 0.1) | 0.144 | 0.0 (-0.0 to 0.0) | 0.433 | 0.0 (0.0 to 0.0) | 0.200 | **0.2 (0.1 to 0.3)** | **0.001** |
| **1-year timepoint** | **-3.5 (-5.0 to -1.9)** | **<0.001** | **-3.1 (-4.2 to -1.9)** | **<0.001** | **-2.0 (-2.7 to -1.3)** | **<0.001** | **-13.5 (-18.8 to -8.2)** | **<0.001** |
| **2-year timepoint** | **-4.4 (-6.3 to -2.5)** | **<0.001** | **-3.6 (-5.0 to -2.2)** | **<0.001** | **-3.0 (-3.9 to -2.2)** | **<0.001** | **-10.9 (-17.2 to -4.6)** | **<0.001** |

95%CI: 95% Confidence Interval; ESR: erythrocyte sedimentation rate; SJC: Swollen Joints Count; TJC: Tender Joints Count; PGH: Patient Global Health;
