# Supplementary material for: Impact of multimorbidity on the first ts/bDMARD effectiveness and retention rate after two years of follow-up in patients with rheumatoid arthritis from the BIOBADASER registry
Source: Arthritis Res Ther. 2024 Feb 23;26:57. doi: 10.1186/s13075-024-03287-9 (PMC10885598; doi:10.1186/s13075-024-03287-9)
Supplement: Supplementary file 2 — Supplementary Material 2. [file 13075_2024_3287_MOESM2_ESM.docx]

**Supplementary Table 2. Association between the Charlson Comorbidity Index score as continuous variable and the change in the DAS28 and their components.**

|  | **Adjusted linear regression**  **DAS28** | | **Adjusted linear regression**  **TJC** | | **Adjusted linear regression**  **SJC** | | **Adjusted linear regression**  **PGH** | | **Adjusted linear regression**  **ESR** | |
| --- | --- | --- | --- | --- | --- | --- | --- | --- | --- | --- |
|  | **Beta coefficient (95%CI)** | **p-value** | **Beta coefficient (95%CI)** | **p-value** | **Beta coefficient (95%CI)** | **p-value** | **Beta coefficient (95%CI)** | **p-value** | **Beta coefficient (95%CI)** | **p-value** |
| **Multimorbidity (CCI score)** | **0.1 (0.0 to 0.2)** | **0.011** | -0.0 (-0.5 to 0.4) | 0.890 | **0.5 (0.2 to 0.8)** | **0.003** | -0.0 (-0.2 to 0.2) | 0.814 | **4.3 (2.5 to 6.1)** | **<0.001** |
| **Sex (female)** | **0.4 (0.2 to 0.5)** | **<0.001** | 0.8 (-0.0 to 1.6) | 0.060 | **0.0 (-0.6 to 0.7)** | 0.933 | **0.4 (0.0 to 0.7)** | **0.034** | **5.1 (1.7 to 8.5)** | **0.003** |
| **Age at the drug initiation** | **0.0 (0.0 to 0.0)** | **0.002** | 0.0 (-0.0 to 0.1) | 0.156 | 0.0 (-0.0 to 0.0) | 0.377 | 0.0 (0.0 to 0.0) | 0.237 | **0.2 (0.1 to 0.3)** | **0.002** |
| **1-year timepoint** | **-1.5 (-1.8 to -1.1)** | **<0.001** | **-3.5 (-5.0 to -1.9)** | **<0.001** | **-3.0 (-4.2 to -1.9)** | **<0.001** | **-2.0 (-2.7 to -1.3)** | **<0.001** | **-13.5 (-18.8 to -8.3)** | **<0.001** |
| **2-year timepoint** | **-1.8 (-2.2 to -1.4)** | **<0.001** | **-4.4 (-6.3 to -2.5)** | **<0.001** | **-3.6 (-5.1 to -2.2)** | **<0.001** | **-3.0 (-3.9 to -2.2)** | **<0.001** | **-11.0 (-17.3 to -4.7)** | **0.001** |

95%CI: 95% Confidence Interval; ESR: erythrocyte sedimentation rate; SJC: Swollen Joints Count; TJC: Tender Joints Count; PGH: Patient Global Health;
